# From *ex ovo* to *in vitro*: xenotransplantation and vascularization of mouse embryonic kidneys in a microfluidic chip†

**DOI:** 10.1039/d4lc00547c

**Published:** 2024-09-02

**Authors:** Micaela Oliveira, Partha Protim Sarker, Ilya Skovorodkin, Ali Kalantarifard, Tugce Haskavuk, Jonatan Mac Intyre, Elizabath Nallukunnel Raju, Samin Nooranian, Hiroki Shioda, Masaki Nishikawa, Yasuyuki Sakai, Seppo J. Vainio, Caglar Elbuken, Irina Raykhel

**Affiliations:** a Microfluidics and Biosensor Research Group, Disease Networks Research Unit, Department of Biochemistry and Molecular Medicine, University of Oulu Finland caglar.elbuken@oulu.fi; b VTT Technical Research Centre of Finland Ltd. Finland; c Developmental Biology Laboratory, Disease Networks Research Unit, Faculty of Biochemistry and Molecular Medicine, University of Oulu Oulu Finland seppo.vainio@oulu.fi irina.raykhel@oulu.fi; d Laboratory of Organs and Biosystems Engineering, Department of Chemical System Engineering, University of Tokyo Tokyo Japan; e Infotech Oulu, University of Oulu Oulu Finland; f Kvantum Institute, University of Oulu Oulu Finland

## Abstract

Organoids are emerging as a powerful tool to investigate complex biological structures *in vitro*. Vascularization of organoids is crucial to recapitulate the morphology and function of the represented human organ, especially in the case of the kidney, whose primary function of blood filtration is closely associated with blood circulation. Current *in vitro* microfluidic approaches have only provided initial vascularization of kidney organoids, whereas *in vivo* transplantation to animal models is problematic due to ethical problems, with the exception of xenotransplantation onto a chicken chorioallantoic membrane (CAM). Although CAM can serve as a good environment for vascularization, it can only be used for a fixed length of time, limited by development of the embryo. Here, we propose a novel lab on a chip design that allows organoids of different origin to be cultured and vascularized on a CAM, as well as to be transferred to *in vitro* conditions when required. Mouse embryonic kidneys cultured on the CAM showed enhanced vascularization by intrinsic endothelial cells, and made connections with the chicken vasculature, as evidenced by blood flowing through them. After the chips were transferred to *in vitro* conditions, the vasculature inside the organoids was successfully maintained. To our knowledge, this is the first demonstration of the combination of *in vivo* and *in vitro* approaches applied to microfluidic chip design.

## Abstract 2

The new lab-on-a-chip design allows organoids of different origin to be cultured and vascularized on CAM and to be transferred them *in vitro* conditions.

## Graphical abstract

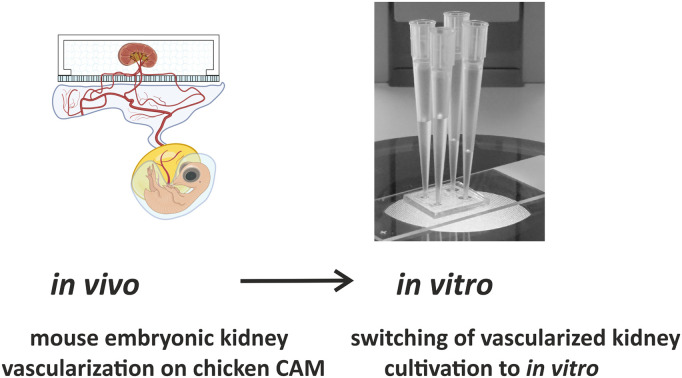

## 1. Introduction

Microfluidic systems recapitulating the blood flow and organization of the vasculature *in vitro* are rapidly evolving as indispensable tools to study complex biological structures. First started as an approach to study the peculiarities of microvasculature development,^1–3^ they are now being directed towards mimicking the biology of multicellular clusters, organoids, mini tissues and even the whole organism in miniature.^4–6^ Cell biology arose as a scientific discipline from the discovery of the fact that the individual cells of multicellular organisms can be cultured and propagated *in vitro*. Further evolution of this idea can be linked to the generation of organoids – miniaturized multicellular structures representing the corresponding organs in morphology and function.^7–9^ Recently, we have been witnessing the increase of complexity of *in vitro* biological systems, as the multicellular living systems in microcirculation conditions resemble the endothelial vasculature in living organisms.^10^ The blood circulation system, which is considered to be a specific tissue in multicellular organisms, maintains many important functions such as gas exchange, nutrition delivery, distribution of hormones and other long-range signals, *etc.* Thus, it is of utmost importance to add the microcirculation/vascularization modality to tissue/organ-on-a-chip experiments designed for the needs of pharmacology, toxicology and general medical biology. A number of different types of organoids, including lung, liver, pancreas, skin, heart, intestine, brain, prostate, retina and tumours, have been successfully vascularized *in vitro* on microfluidic chips.^11,12^ However, only the initial steps of kidney organoid vascularization have been observed on a chip.^13–15^ More importantly, providing microcirculation conditions for kidney organoid cultures is critical, since the main function of the kidney is blood filtration. In the absence of blood flow (or at least a culture medium perfusion), this function cannot be reproduced *in vitro*. Taking these facts into account, we have identified *in vitro* kidney vascularization as an ideal model and chose the mammalian embryonic kidney as the object of our research. Amongst the many microfluidic *in vitro* vasculature systems that have been published so far, the “three lane design” has become the most popular.^16,17^ In this microfluidic system, the central lane is loaded with a hydrogel and lateral channels provide a perfusion option.^18–22^ Typically, endothelial cells and supporting mesenchymal cells are loaded to lateral channels, mimicking angiogenesis, or to the central channel, reproducing vasculogenesis. As a result, a perfusable network of *in vitro* generated blood vessels is formed in the hydrogel, recapitulating the capillary plexus.^18,21,23,24^

In most *in vitro* vasculature related publications, the sources of endothelial cells were primary human umbilical vein cells (HUVECs).^25,26^ The advantage of these cells is that even in the simplest experimental scheme (*i.e.*, without supporting cells) they form a network of hollow tubules inside the hydrogel.^27,28^ At the same time, these tubular structures are rather aberrant and do not closely recapitulate *in vivo* vasculature at the morphological and physiological levels.^29,30^ This can be explained by the fact that HUVECs have a specific function in the umbilical cord and are never found elsewhere *in vivo*. The negative effects of HUVECs on kidney organoids and *vice versa* have been previously reported.^13^ Thus, to achieve *in vitro* vascularization to kidney organoids, we need to find alternative cell types.^31,32^

*In vivo* transplantation has been proposed as an option for *in vitro* vascularization.^15,33–43^ However, this approach is very laborious, time consuming, costly, and does conform with RRR standard requirements.^44–47^ Exceptionally, xenotransplantation to a chicken embryo can be a viable solution. Currently, there is no ethical concern with using chicken embryos until day 16 of development; thus, they are do not fall under RRR requirements. Xenotransplantation to chicken chorioallantoic membrane (CAM) is a very popular and widely applied assay.^15,48–53^ It has also been previously shown that many different types of cells and tissue samples induce the sprouting of chicken blood vessels.^50,54–57^ For example, in several studies, it was shown that the mouse embryonic kidney “attracts” chicken blood vessels as a result of xenotransplantation.^15,51–53,58–61^

It should be noted that the CAM assay also has its limitations. It has a relatively short time window, around 5–6 days, between the sufficient development of the CAM for transplantation and the point at which the experiment should be terminated for ethical reasons. To address this issue, we propose a methodology in which the vascularization of the recipient tissue/organoid is started on CAM *via* xenotransplantation and then continued *in vitro*.

In this article, we present a novel flexible microfluidic assembly that can be installed on the chicken CAM conformally, from which blood vessels penetrate into the chamber, organize the vascular system and vascularize the mouse embryonic kidney. This unique design allowed the system to be later transferred from the CAM to an *in vitro* system and continue to grow for several days. This method can be applied by other organ/organoid-on-a-chip systems to address the necrotic core problem due to the lack of or poor vascularization. The microfluidic chips presented in this work can be scaled to host multiple organoids, and several microfluidic chips can be placed on the same chicken CAM to vascularize different types of organoids with varying perfusion or culture conditions.

## 2. Experimental section

### 2.1. Materials and reagents

Polydimethylsiloxane (PDMS) and curing agent (Sylgard 184™ Silicone Elastomer Kit, Dow Corning); ARseal™ (Adhesives Research); ARcare® 90106NB (Adhesives Research); polyester porous membrane (PETE) (Sterlitech Corp., PET8047100); phosphate buffered saline (PBS) (Sigma Aldrich); Dulbecco's modified Eagle's medium (DMEM) + GlutamaxTM (5.56 mM glucose, 1 mM pyruvate) (Gibco); fetal bovine serum (FBS) (HyClone); penicillin–streptomycin (Sigma Aldrich); fibrinogen (Sigma Aldrich); vascular endothelial growth factor (VEGF) (Sigma Aldrich); aprotinin (Sigma Aldrich); thrombin (Sigma Aldrich); paraformaldehyde (PFA) (Sigma Aldrich); primary antibody CD31 (Purified Rat Anti-Mouse CD31; 1 : 100, BD Pharmingen™), Hoechst (1 : 1000; Cat # 62249, ThermoFisher Scientific, United States); secondary antibody goat anti-rabbit Alexa Fluor 647 (1 : 1000; Cat # A21244, ThermoFisher Scientific, United States); bovine serum albumin (BSA); Triton X-100 (Cat # T8787, Sigma Aldrich, United States); fertilized avian eggs (Haaviston Siitoskanala, Panelia, Finland); wild type (WT) embryonic kidneys (Charles River Laboratories).

### 2.2. Fabrication

#### Mould design

**Fig. 1:**
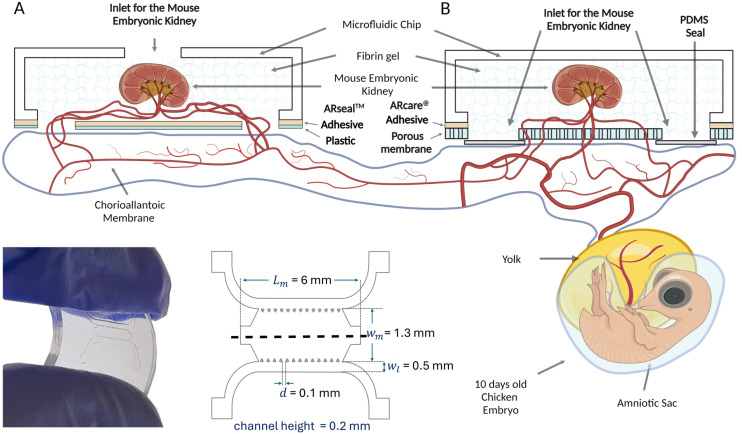
Schematic representation of the microfluidic chip on a CAM. Prototypes A and B are shown in lateral and top view with dimensions. The mouse embryonic kidney is schematically vascularized by capillaries originating from the chick embryo chorioallantoic membrane (CAM). Created with https://BioRender.com.

The mould for the fabrication of the microfluidic chambers was generated using standard soft lithography technology using SU-82100 according to the manufacturer's recipe for a thickness of 200 μm. To fabricate the microfluidic device, Sylgard 184 (Dow Corning) polydimethylsiloxane (PDMS) was used at a 10 : 1 ratio of base polymer to curing agent. The degassed mixture was poured on the mould to a thickness of 3 mm and cured by baking at 80 °C for 2 hours. Once cooled, the PDMS was carefully peeled off the wafer (Fig. 1) and cut into microfluidic chips. Due to the very small size of the chip, we obtained 20 microfluidic chips using a 100 mm diameter silicon wafer mould. The PDMS microfluidic device is based on the classical three lane microfluidic chip design. The middle channel was intended for loading of the hydrogel and embryonic kidneys subjected to vascularization. The lateral channels provide for the flow of culture medium during perfusion of the vascularized tissue samples *in vitro*. The dimensions of the chip are indicated in Fig. 1.

#### Microfluidic chip design

A microfluidic chip suitable for transplantation to a chicken embryo should be fabricated from non-toxic, biologically compatible materials and be as light as possible to avoid mechanical damage of the delicate CAM membrane. Additionally, the flexibility of the chip is an important feature to allow it to conform to the curved surface of the biological structure. We tested several types of materials and chamber designs and eventually used a composite assembly of PDMS and plastic connected by an adhesive layer of ARseal™ or ARcare® material (Adhesives Research). Traditional plasma bonding was found to be inefficient for connecting the PDMS to the plastic or polyester porous membranes.

Two prototypes of the microfluidic chamber were generated (Fig. 1). In the first one, the chicken derived vasculature grew into the chip through two 1 mm diameter openings in the ARseal™ plastic membrane (Fig. 1A). In the second prototype, the ARseal™ membrane was replaced with a polyester porous membrane (Sterlitech Corp., PET8047100) and the chicken vasculature grew through 30 μm pores (Fig. 1B).

#### Assembly of the chips

**Fig. 2:**
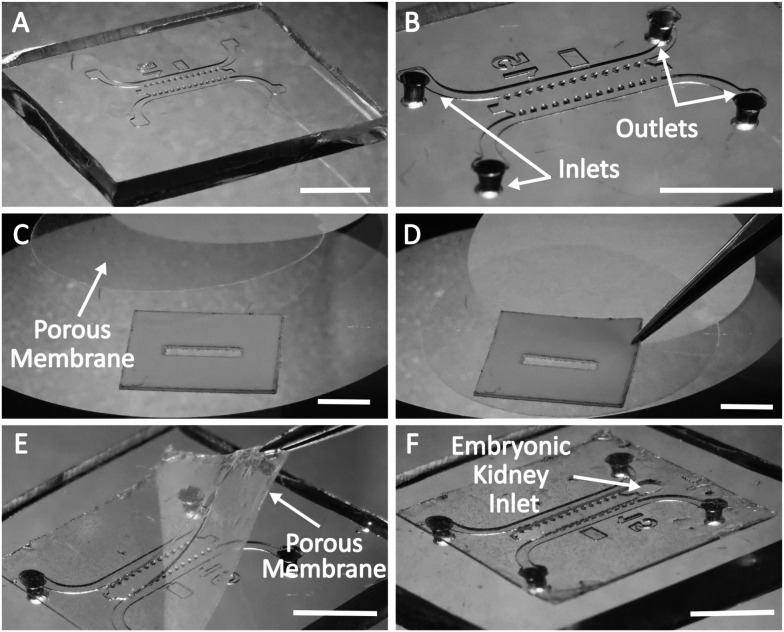
Illustration of the assembly of the microfluidic chip of prototype B. A. The PDMS microfluidic chip produced using a silicon/SU8 mould. B. After punching the holes for loading culture medium into the lateral channels using a 1 mm skin biopsy puncher. C. After attaching the porous membrane to ARcare®. D. After removal of the upper layer from ARcare® and attachment of the porous membrane. E. After attaching the porous membrane with ARcare® to the microfluidic chamber. F. The microfluidic chip is ready for loading. Scale bars: 5 mm.

The assembly of the microfluidic chamber of the prototype B is represented in Fig. 2. First, the microfluidic block was cut from a PDMS replica (Fig. 2A). Then, inlets for lateral channels were punched using a 1 mm skin biopsy puncher (Fig. 2B). A blank of the adhesive layer of the chip was cut from ARcare® using a laser cutter (Flux Beamo 30 W). The upper liner of ARcare® was replaced with a porous membrane (Fig. 2C and D), which was subsequently cut to the correct size with scissors. Two holes of 1 mm diameter were punched at the ends of the middle channel with a skin biopsy puncher. Then, the release liner was removed from the ARcare®, and the blank was aligned and assembled with the PDMS block under a stereomicroscope (Fig. 2E). After disinfection of the chip with UV light under the hood (15 min for each side), the chip was ready for loading (Fig. 2F).

Fabrication of prototype A was performed in essentially the same way; however, ARseal™ was used instead of ARcare® and an additional 1 mm diameter hole was punched in the center of the middle channel of the PDMS block with a skin biopsy puncher. This opening served as a “port” for inserting the embryonic kidney sample into the chamber.

Prototype A was intended only to provide evidence of vascularization of mouse embryonic kidney inside the chip placed on the CAM. Further transferring of this chip design to *in vitro* conditions was not planned. In prototype B, the additional hole at the top of the chamber would need to be sealed when the chip was transferred from CAM to *in vitro* culture. To avoid this extra step (and potential risks of leakage), we loaded the kidneys onto the chip from below, along with the gel through the loading holes in the porous membrane. Although this way of loading the kidneys onto the chip requires some hands-on practice, it prevents any complications due to top port leakage and ensures hassle-free organoid observation both *ex ovo* and *in vitro*.

### 2.3. Loading of the chips

**Fig. 3:**
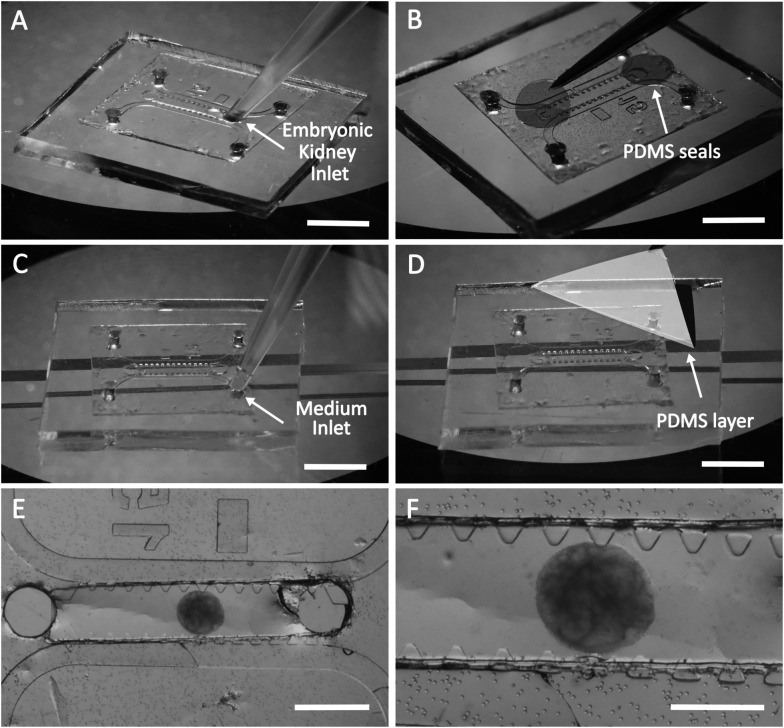
Loading of the microfluidic chip with the hydrogel, mouse embryonic kidneys and culture medium. A. Loading of the embryonic kidney in fibrin gel into the central channel. B. Loading of the cell culture medium into the lateral channels. C. Attachment of the PDMS thin layer to the chip. D. Attachment of the PDMS round patches to the loading ports. E. General overview of the prototype B microfluidic chip with a mouse embryonic kidney in fibrin gel. F. The mouse embryonic kidney on the porous membrane with holes of 30 μm is shown at higher magnification. Scale bars for A–D: 5 mm; for E: 1 mm; for F: 500 μm.

Loading of the microfluidic chip (prototype B) prior to transplantation to the chicken embryo is illustrated on Fig. 3.

First, the microfluidic chip was positioned with the porous membrane up and the PDMS block down (Fig. 3A). Fibrin gel was prepared as previously reported.^62^ A solution of fibrinogen (10 mg ml^−1^) supplemented with 0.45 U ml^−1^ aprotinin and 50 ng ml^−1^ VEGF was prepared in phosphate-buffered saline to yield a final fibrinogen concentration of 2.5 mg ml^−1^. VEGF was applied to induce angiogenesis, and aprotinin reduced the gel degradation caused by proteases excreted by the mouse and chicken-derived cells.^63^ Thrombin was then added to a final concentration of 1 U ml^−1^, and the solution containing the embryonic kidney was immediately loaded into the middle channel using a micropipette (Fig. 3A). After loading of the gel, the holes at the ends of the middle channel were sealed with thin (up to 100 μm) PDMS patches (Fig. 3B). At this step, the chip was placed in a cell incubator in a moist chamber for 10–15 minutes to let the gel polymerise.

For the next step, *i.e.*, loading of the lateral channels with culture medium, the chip should be turned upside down (Fig. 3C). To avoid contact with the membrane and deformation of the gel, the chip was organized on a special support (Fig. 3C). As a support, we used two object glasses for light microscopy attached to a plastic Petri dish 5 mm apart. After placement of the chip on the support, the lateral channels were loaded with culture medium (Fig. 3C), and the openings of the lateral channels were sealed with a thin layer of PDMS (Fig. 3D). As a result, the composite microfluidic chip was loaded in the middle channel with fibrin hydrogel containing mouse embryonic kidney and in the lateral channels with culture medium supporting the viability of the kidney before the chicken-derived blood vessels grow into the chip (Fig. 3E and F). The microfluidic chip was sealed with thin PDMS patches, except the pores of the membrane facing the CAM.

### 2.4. Transferring of the chip to the chicken CAM

**Fig. 4:**
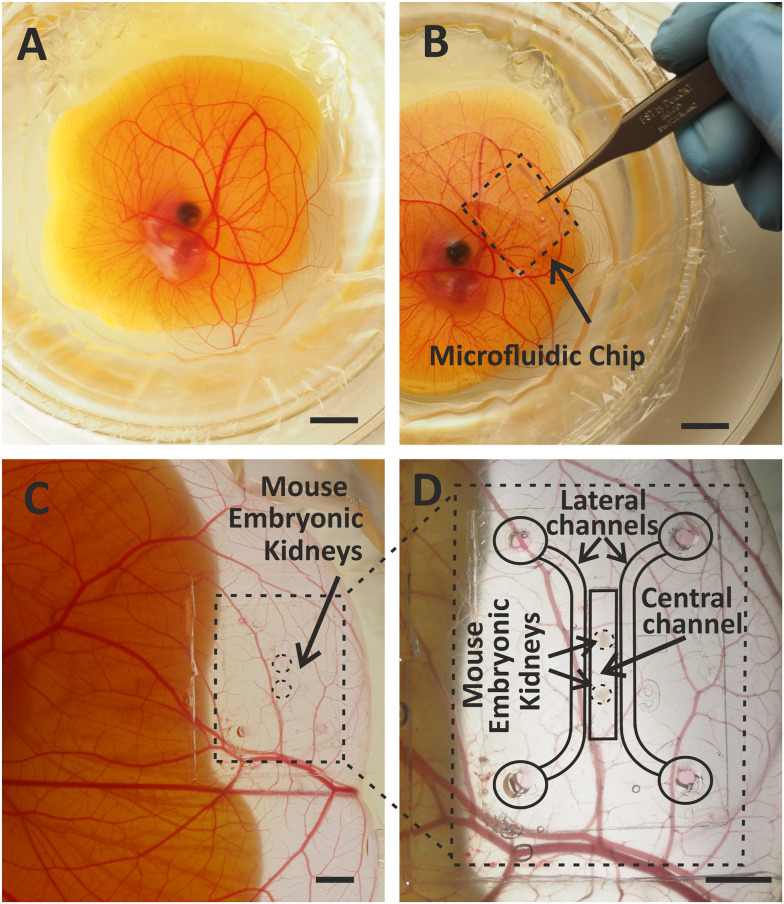
Vascularization of the embryonic kidney in a microfluidic chip on the chicken CAM *ex ovo*. A. A chicken embryo in *ex ovo* culture. B. Embryonic kidney in a microfluidic chip on the chicken CAM. C. and D. Vascularized embryonic kidney in a microfluidic chip on the chicken CAM (C) and at higher magnification with schematically marked parts (D). Scale bars: 10 mm.

Assembled and loaded microfluidic chip was transferred to the chicken CAM with the porous membrane facing down (Fig. 4A and B). The best spot for transplantation of the chip was further away from the chicken embryo and in the area of the CAM lacking large blood vessels. Placing the microfluidic chip directly on a large artery or vein might disturb the blood flow and reduce the viability of the chicken embryo (Fig. 4C and D).

**Fig. 5:**
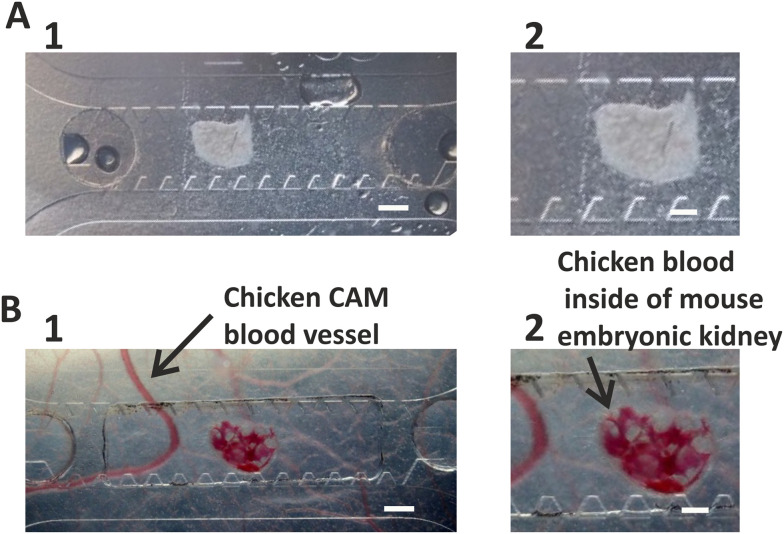
Vascularization of mouse embryonic kidney inside the microfluidic chip transplanted onto the CAM. A. Mouse embryonic kidney loaded into the microfluidic chip before transplantation onto the CAM. B. Mouse embryonic kidney vascularized as a result of transplantation on CAM 4 days after transplantation. A large chicken blood vessel and chicken blood inside the mouse embryonic kidney are indicated with arrows. Scale bars for A1, B1: 500 μm; for A2, B2: 200 μm.

The microfluidic chip was transplanted onto the chicken CAM at day 9 of embryonic development (Fig. 5A) and transferred to *in vitro* culture at day 13–14, when the vascularization of the kidneys in the chip occurred (Fig. 5B).

### 2.5. Transferring of the chip from the chicken CAM to *in vitro* culture

**Fig. 6:**
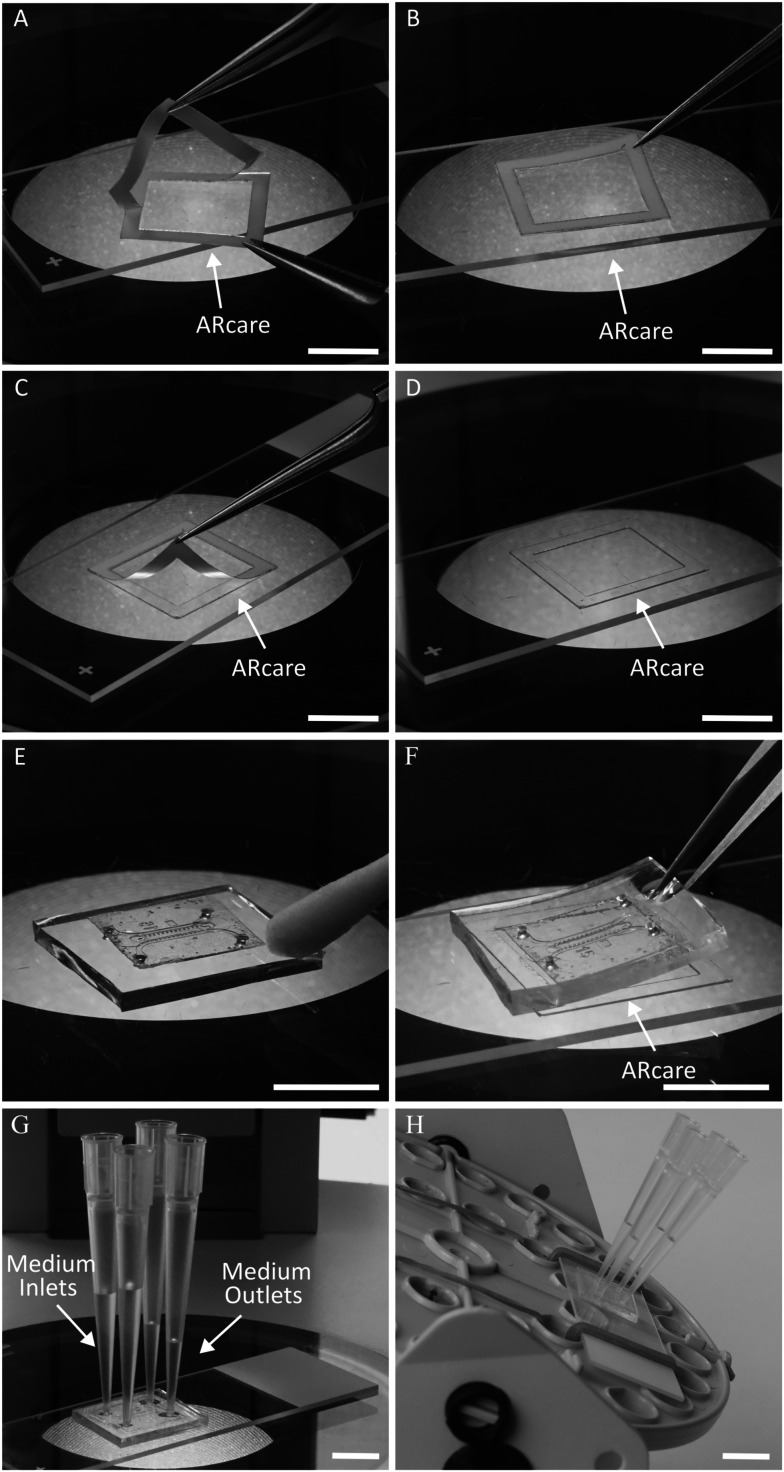
Assembly of the microfluidic chip with vascularized embryonic kidney for cultivation *in vitro*. A. Removing the first release layer from ARcare®. B. Mounting of the ARcare® on the object glass. C. Removing the second release layer from ARcare®. D. Adhesive layer assembled on the object glass. E. Cleaning of CAM tissue residuals from the surface of the chip. F. Flipping the microfluidic chip and attaching it to the microscopic glass slide. G. Placing four pipette tips for perfusion through the lateral channels of the microfluidic chips. H. Assembly of the chip on the rocking platform.

After the vascularization of the mouse embryonic kidney transplanted to the CAM inside the chip was confirmed by light microscopy (Fig. 5), the chip was transferred into *in vitro* culture conditions. First, the blank of the adhesive layer was cut from the ARcare® using a laser cutter. Then, the adhesive layer was assembled on an object glass (Fig. 6A–D). The microfluidic chip was carefully removed from the CAM with forceps and placed under a dissection stereomicroscope with the porous membrane up. The edges of the PDMS block were cleaned of residuals of chicken tissue with cotton or filter paper (Fig. 6E). Then, the chip was flipped and attached to the adhesive layer under the stereomicroscope (Fig. 6F). Firm attachment of the PDMS to the glass was confirmed under the microscope. The thin PDMS membrane sealing the inlets of the lateral channels was removed from the upper side of the chip and four reservoirs filled with culture medium were attached to the chip (Fig. 6G). The assembled chip was placed on a custom-designed rocking platform changing the position in 90° every 20 min (Fig. 6H). The microfluidic chip was kept in these conditions for 4 days. The culture medium in the reservoirs was replaced with fresh medium daily. At the end of the experiment, the microfluidic chamber was carefully detached from the object glass, washed with PBS and fixed with 4% PFA.

### 2.6. Cultivation of the embryonic kidneys inside the microfluidic chip exclusively *in vitro*

As a control for the experiments described above, we cultured mouse embryonic kidneys transferred into microfluidic chambers solely *in vitro* (without transplanting onto CAM). Microfluidic chips were loaded with hydrogel and embryonic kidneys essentially in the same way as described in 2.3. Then, the chips were arranged on object glasses as described in 2.5 (Fig. 6A–D and F–H).

The microfluidic chip was kept under these conditions for 4 days. The culture medium in the reservoirs was replaced with fresh medium daily.

At the end of experiment, the microfluidic chamber was carefully detached from the object glass, washed with PBS and fixed with 4% PFA.

### 2.7. Immunostaining

Mouse embryonic kidneys immediately after dissection and inside the microfluidic chips were fixed with 4% PFA for 30 min, washed three times with PBS, blocked for 3 h with blocking buffer containing 1% BSA, 0.1% Triton X-100 (Cat # T8787, Sigma Aldrich, United States), 10% FBS and 10% goat serum in PBS at room temperature. Then, the mouse embryonic kidneys were incubated with primary antibodies CD31 (Purified Rat Anti-Mouse CD31; 1 : 100, BD Pharmingen™) overnight at 4 °C. After washing with blocking buffer, the embryonic kidneys were incubated with Hoechst (1 : 1000; Cat # 62249, ThermoFisher Scientifc, United States) and secondary antibodies goat anti-rabbit Alexa Fluor 647 (1 : 1000; Cat # A21244, ThermoFisher Scientific, United States). After washing with PBS, the embryonic kidneys were mounted using Immu Mount (ThermoScientific, United States).

### 2.8. Light microscopy

Bright-field and fluorescence imaging of the mouse embryonic kidneys in the microfluidic chip transplanted onto the chicken embryonic CAM was done using an Olympus SZ40 stereo microscope with an Olympus OM-D E-M10 Mark II camera and Zeiss Axio Zoom.V16 fluorescence stereo microscope. Confocal imaging of the mouse embryonic kidneys immediately after dissection and in the microfluidic chip at different stages of the experiment was done using a Zeiss LSM 780 laser scanning confocal microscope with Zen Black software. The images were post-processed using Zeiss Zen Blue software.

### 2.9. Setting up *ex ovo* chicken culture

Chicken embryonic culture *ex ovo* were grown as described previously.^52,64,65^ Briefly, a fertilized egg stored at +14 °C was incubated for 72 hours at 37.5 °C in a humidified egg incubator (Grumbach GmbH) on an automatic egg turner (OLBA B.V.). Then, the egg was opened and entire contents were transferred to a water bed chamber. The chicken embryo was cultivated for 6–7 days in a cell culture incubator (+37.0 °C, 90% humidity, 5% CO2). At this stage, the chicken embryo was developed enough that the CAM could be used for transplantation.

### 2.10. Dissection of mouse embryonic kidneys and setting up organotypic kidney cultures

In our study, animal care and experimental procedures were performed in accordance with the Finnish national legislation for laboratory animals, EU Directive 86/609/EEC and European Convention for the protection of vertebrate animal used for experimental and other scientific purposes (ETS 123) and the EU Directive 2010/63/EU. Mice used for the experiments were authorized by the National Animal Experiment Board Finland (ELLA) and the Oulu Laboratory Animal Center of University of Oulu. Mice were obtained from the Oulu Laboratory Animal Center, where they were housed in bedding with environmental enrichments, and were given unrestricted access to standard rodent chow and water. Wild type (WT) embryonic kidneys were used in our study. Pregnant outbred WT CD1 (Charles River Laboratories) mice were used as a source of WT embryonic kidneys. The CD1 mouse license 17/2021 has been described previously.^66^

Mouse embryonic kidneys were isolated as described previously.^67^ Mouse embryonic kidneys *in vitro* were cultivated in a culture incubator (+37.0 °C, 5% CO_2_) in Dulbecco's modified Eagle's medium (DMEM) + GlutamaxTM (5.56 mM glucose, 1 mM pyruvate) (Gibco) supplemented with 10% fetal bovine serum (FBS) (HyClone) and 0.5% penicillin–streptomycin (Sigma).

## 3. Results and discussion

### 3.1. Overview of microfluidic chip model

To implement the idea of organ vascularization in a microfluidic chip in an *in vivo* system and subsequent transfer of this chip to *in vitro* conditions, two prototypes were tested. In prototype A (Fig. 1A), the ARseal™ membrane was located at the bottom of the chamber facing the chicken CAM. Blood vessels of chicken embryo grew into the hydrogel through the two ports in the ARseal™ membrane (Fig. 1A).

Prototype A served as a proof of concept to confirm the growth of chicken-derived blood vessels into the hydrogel and vascularization of the mouse embryonic kidney by the chicken vasculature inside of the microfluidic chip transplanted onto the CAM (Fig. S1, ESI† Videos S1 and S2). However, during the next step of the experiment, *i.e.*, detaching the chip from the chicken CAM, the hydrogel and chicken blood vessels grown into the chip were severely damaged. In most cases, the section above the 1 mm holes in the plastic was ripped off the chip and stayed with the CAM. Thus, transferring the chip of this design to *in vitro* conditions was not possible.

Prototype B (Fig. 1B) was designed to improve the transfer of the chip from *in vivo* to *in vitro* culture. In this design, chicken-derived blood vessels grew through the 30 μm pores of the membrane located at the bottom of the chip (Fig. 1B and 5). During the detachment of the chip from the CAM, the chicken-derived capillaries were ripped, but the hydrogel and mouse embryonic kidney in the chip remained intact. Thus, the membrane provided a strong mechanical support for the hydrogel and prevented it from being damaged during the transfer of the chip from the *ex ovo* to *in vitro* culture.

### 3.2. Vascularization *in vivo* and transfer to *in vitro* conditions

Essentially any tissue sample of a suitable size will be vascularized as a result of xenotransplantation to chicken CAM.^50,68–71^

Chicken-derived blood vessels might grow inside the transplanted tissue or fuse with existing recipient-derived vasculature forming chimerical blood vessels.^72^ Many factors may stimulate the directed growth of chicken blood vessels; however, the main one is thought to be the gradient of VEGF. To attract the sprouting of CAM-derived vasculature towards the mouse embryonic kidney, we added VEGF to the hydrogel inside the microfluidic chip. It is known that the embryonic kidney itself also generates VEGF,^73^ which further promotes migration of chicken blood vessels into the microfluidic chamber transplanted onto the CAM.

Previously, we presented the application of minichambers for efficient vascularization of mouse embryonic kidneys and kidney organoids.^52^ However, these chambers did not provide an option for continuation of the experiment *in vitro* after initial vascularization *in vivo*. With the novel design of microfluidic chamber presented in this paper, we have demonstrated that blood vessels can grow into the hydrogel and successfully vascularize the tissue sample located inside the chip.

The first blood vessels originating from chicken embryo were detected in the chamber 3–4 days after placing it onto the CAM. Vascularization of mouse embryonic kidney within the chamber occurred 3–5 days after placement of the chip onto CAM and was be confirmed by the blood flow inside it (ESI† Videos S3).

Transferring the microfluidic chamber from the CAM to *in vitro* culture is the most critical step in the assay procedure. Special attention to cleaning the edges of PDMS block is required. If the PDMS surface is not completely dry and contains residues of chicken embryo tissue, the chamber is not sealed with the adhesive layer, and leakage of the culture medium is observed during further cultivation *in vitro*.

### 3.3. Analysis of the vasculature

**Fig. 7:**
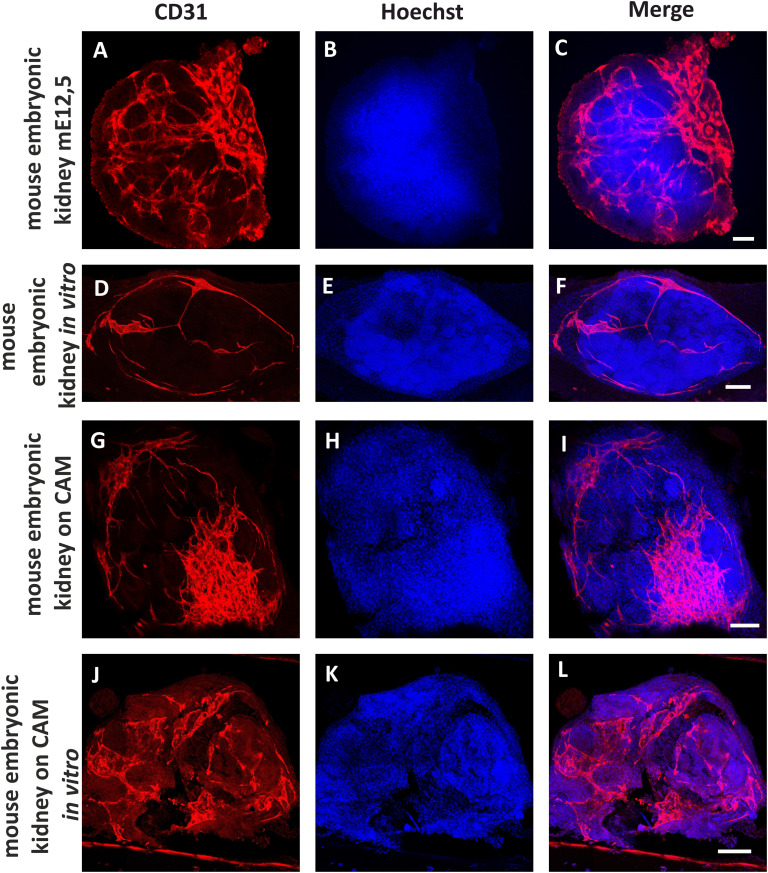
Detection of the blood vessels in mouse embryonic kidneys in different variants of the experiment. Positive control: intact mouse embryonic kidney dissected from E12,5 embryo (A–C). Negative control: mouse embryonic kidney dissected from E12,5 embryo, cultivated 5 days *in vitro* (D–F). *In vivo* experiment. Mouse embryonic kidney E12,5 was vascularized on a chicken embryo CAM for 4–5 days (G–I). *In vivo* experiment after continuation *in vitro*. Mouse embryonic kidney E12,5 was vascularized on the chicken CAM for 4–5 days, and then cultivated 5 days under *in vitro* conditions (J–L). CD31 was used as an endothelial marker (red) and Hoechst as a nuclear marker (blue). Scale bars = 50 μm.

The general aim of the presented research was to design a microfluidic chamber in which tissue samples can be vascularized by chicken embryo CAM and the experiment can be continued *in vitro* without significant degradation of the sample. Therefore, we compared the development of blood vessels in mouse embryonic kidneys placed in microfluidic chambers under three different conditions: 1. *in vitro*; 2. transplanted onto CAM; 3. transplanted onto CAM and then transferred to *in vitro* conditions, using light microscopy of living samples during the experiment as well as immunostaining and confocal microscopy of fixed samples (Fig. 7; quantification of vascular density is represented in ESI† Fig. S2). All experiments were repeated at least three times. The survival rate of the chicken embryos in *ex ovo* cultures was 67% (*N* = 9). The success rate of vascularization of the mouse embryonic kidneys on the CAM was 67% (*N* = 6).

As a positive control we used an mE15,5 mouse embryonic kidney fixed immediately after dissection (Fig. 7A–C). The distribution of the kidney endothelial cells in this case corresponds to published data.^13,73–77^

Mouse embryonic kidneys cultivated in the microfluidic chambers of Prototype B exclusively *in vitro* (without transplantation to CAM) served as a negative control (Fig. 7D–F). After 5 days of cultivation in culture medium inside the microfluidic chamber, the vasculature network was drastically reduced; however, individual endothelial cells were still present in the kidney. These data are in accordance with previous reports in which it was shown that organotypic culture conditions significantly inhibit development of kidney vasculature but do not eliminate endothelial cells completely.^73^

When microfluidic chamber containing mouse embryonic kidneys was transplanted onto the CAM, the chicken-embryo-derived blood vessels that grew into the chip fused with mouse-derived capillaries (Fig. 7G–I). Thus, blood circulation was restored in the mouse kidney and vasculature development continued. These results are similar to previous observations of mouse embryonic kidneys transplanted directly onto CAM.^52,58–60^ In these published works, it was shown that chicken blood vessels can either grow inside the transplanted mouse kidneys and directly interact with the nephrons of the donor,^58,60^ or fuse with the mouse-derived capillaries, providing re-vascularization of the mouse kidney with its own endothelium.^52^

It has been noted in many publications related to *in vitro* vasculature assays that once endothelial cells grown inside of the hydrogel in microfluidic chambers reach the edges of the gel, they open lumen to the space of the lateral channels.^78^ In our experiments, we could see this effect through leakage of chicken blood into the lateral channels of the microfluidic chip during cultivation on the CAM (ESI† Fig. S3). Thus, we can expect that perfusion through the blood vessels formed inside the mouse embryonic kidney can be continued after transferring the chip from the CAM to *in vitro* conditions. For this purpose, we placed the chip on a rocking platform and organized a gradient of hydrostatic pressure between the lateral channels.

Endothelial-cell-specific immunostaining of the samples vascularized on the CAM inside the microfluidic chips (prototype B) and transferred to *in vitro* conditions revealed the presence of intact mouse-derived blood vessels (Fig. 7J–L). Endothelial cells were assembled in the vasculature network, and based on the staining of the cell nuclei (Hoechst staining), the kidney samples did not appear to be severely degraded. Hence, we have confirmed that the vascularization in the chip experiment started on the CAM can be continued under *in vitro* conditions using our custom-designed microfluidic chambers.

## 4. Conclusions

Here, we presented the design, fabrication and application of a composite microfluidic chip consisting of a PDMS replica and porous polyester membrane connected with an adhesive layer. The presented results demonstrated that a tissue sample (mouse embryonic kidney) loaded along with fibrin hydrogel into the chip can be successfully vascularized on chicken CAM and later kept *in vitro* for at least four days.

To our knowledge, this is the first report of a microfluidic device that can be used for initial *in vivo* vascularization of transplanted tissue and later continuation of the perfusion of the sample *in vitro*.

## Data availability

The data supporting this article have been included as part of the ESI.†

## Author contributions

I. S. and I. R. conceived the project. M. O., P. P. S., I. S., A. K., T. H., J. M. I., E. N. R., S. N., H. S. and I. R. designed, performed, and analysed all experimental data. I. R., C. E., S. V., M. N., Y. S. and I. S. supervised the study. M. O. prepared the first draft of the manuscript. C. E., S. V., M. N., Y. S. and I. R., provided the funding for the project. All authors reviewed the manuscript and approved its submission.

## Conflicts of interest

The authors have no conflicts of interest to declare. All co-authors have agreed to the publication of this manuscript.

## Supplementary Material

LC-024-D4LC00547C-s001

LC-024-D4LC00547C-s002

LC-024-D4LC00547C-s003

LC-024-D4LC00547C-s004

LC-024-D4LC00547C-s005

LC-024-D4LC00547C-s006

LC-024-D4LC00547C-s007

LC-024-D4LC00547C-s008
