# Supplementary figures and images for: From ex ovo to in vitro: xenotransplantation and vascularization of mouse embryonic kidneys in a microfluidic chip
Source: Lab Chip. 2024 Sep 2;24(20):4816–26. doi: 10.1039/d4lc00547c (PMC11408908; doi:10.1039/d4lc00547c)

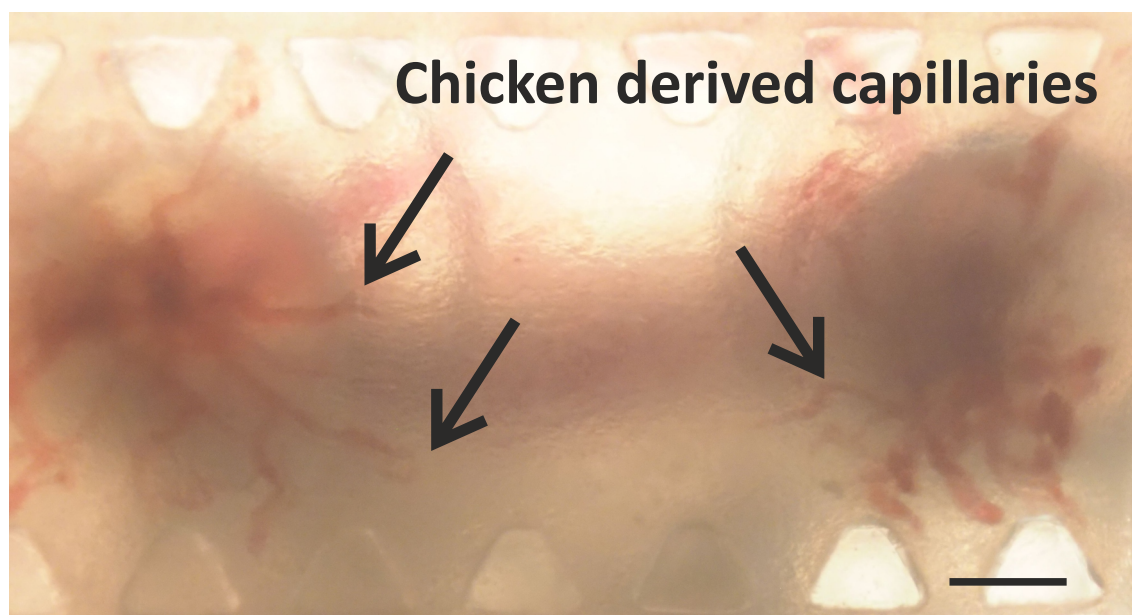

**Suppl. figure 1.**

Supplement: LC-024-D4LC00547C-s002 [file LC-024-D4LC00547C-s002.pdf]

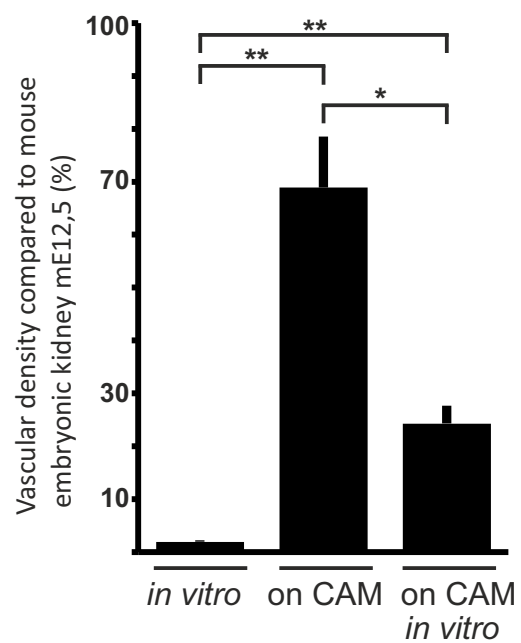

Suppl. figure 2.

Supplement: LC-024-D4LC00547C-s003 [file LC-024-D4LC00547C-s003.pdf]
