# Supplementary material for: From ex ovo to in vitro: xenotransplantation and vascularization of mouse embryonic kidneys in a microfluidic chip
Source: Lab Chip. 2024 Sep 2;24(20):4816–26. doi: 10.1039/d4lc00547c (PMC11408908; doi:10.1039/d4lc00547c)
Supplement: LC-024-D4LC00547C-s004 [file LC-024-D4LC00547C-s004.pdf]

Supplementary Information (SI) for Lab on a Chip.
This journal is © The Royal Society of Chemistry 2024

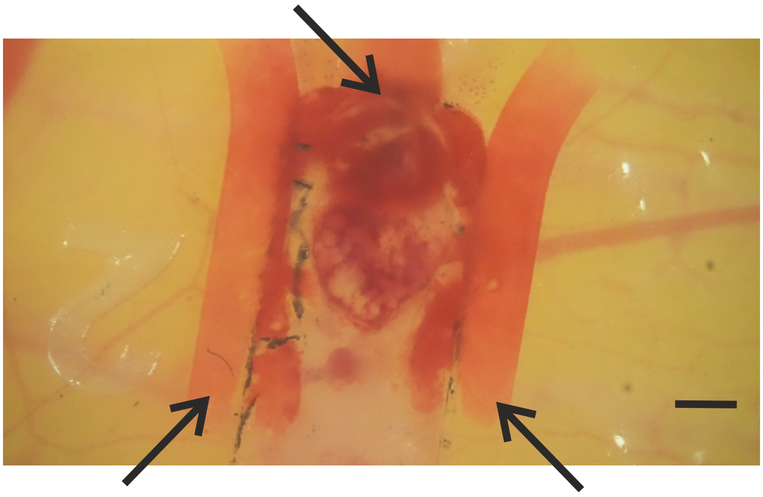

Suppl. figure 3.
