# Supplementary material for: From ex ovo to in vitro: xenotransplantation and vascularization of mouse embryonic kidneys in a microfluidic chip
Source: Lab Chip. 2024 Sep 2;24(20):4816–26. doi: 10.1039/d4lc00547c (PMC11408908; doi:10.1039/d4lc00547c)
Supplement: LC-024-D4LC00547C-s008 [file LC-024-D4LC00547C-s008.pdf]

Supplementary Information (SI) for Lab on a Chip.
This journal is © The Royal Society of Chemistry 2024

Supplementary Figure 1. Chicken derived capillaries (indicated with the arrows) growing into the microfluidic chip on CAM. Scale bar = 200µm.

Supplementary Figure 2. The vascular density of the mouse embryonic kidney on chip, cultivated first on CAM and continued *in vitro* conditions, is higher than that cultivated solely *in vitro*. Vascular density variation in mouse embryonic kidney on CAM, *in vitro* and on CAM and then *in vitro* compared to mouse embryonic kidney mE12,5 (%). Data are presented as mean±s.d., $1<n\leq3$, $*P<0.05$; $**P<0.001$ (Student's t-test for pairwise comparisons).

Supplementary Figure 3. Embryonic chicken blood leaking into the lateral channels of microfluidic chip transplanted to CAM is represented on the photo. Vascularized mouse embryonic kidney inside of the chip and lateral channels with leaked blood are indicated with arrows. Scale bar = 500 µm.

Supplementary video 1. Blood vessels of the chicken embryo grown into the microfluidic chip (Prototype A) transplanted on CAM. Chicken blood flow can be seen in this video.

Supplementary video 2. Vascularization of the mouse embryonic kidney transplanted to CAM inside the microfluidic chip (Prototype A). Chicken blood flow can be seen inside and outside of the mouse embryonic kidney.

Supplementary video 3. Vascularization of the mouse embryonic kidney transplanted to CAM inside the microfluidic chip (Prototype B). Dilated blood vessels inside mouse embryonic kidney are filled with chicken blood. Chicken blood flow also can be seen inside the fine capillaries in mouse embryonic kidney.
